# Harnessing artificial intelligence in plant breeding: innovations in digital phenotyping and breeding methodologies

**DOI:** 10.1007/s00122-026-05293-8

**Published:** 2026-06-23

**Authors:** Nikita Aggarwal, Mukesh Rathore, Farkhandah Jan, Divya Sharma, Sundeep Kumar, Mahendar Thudi, Abdulqader Jighly, Rajeev K. Varshney, Reyazul Rouf Mir

**Affiliations:** 1 Division of Genetics and Plant Breeding, Faculty of Agriculture, Sher-E-Kashmir University of Agricultural Sciences and Technology, Wadura Campus, Sopore, 193201 Kashmir India; 2 Division of Genomics Resources, ICAR-National Bureau of Plant Genetics Resources, New Delhi, India; 3 College of Agriculture, Family Sciences and Technology, Fort Valley State University, Fort Valley, GA USA; 4 AgriSapiens Pty Ltd, Melbourne, VIC Australia; 5 Present Address: WA State Agricultural Biotechnology Centre, Centre for Crop and Food Innovation, Murdoch University, Murdoch, WA 6150 Australia

## Abstract

Agriculture plays a crucial role in the development of countries whose economies rely heavily on food production. In the face of climate change and growing global population, plant breeders are challenged to adopt more efficient crop improvement strategies. The advances in artificial intelligence (AI), particularly in large-scale data integration, analysis, and pattern recognition, have revolutionized several scientific disciplines, including plant breeding. In this review, we provide a comprehensive survey of the potential of AI tools in plant breeding with four key objectives: (i) revolutionizing high-throughput phenotyping, (ii) exploring AI-driven breeding methodologies beyond traditional approaches, (iii) optimizing breeding pipelines through improved modelling of genotype × environment × management interactions, and (iv) highlighting the limitations of AI in plant breeding and future directions. Case studies published during the past two decades illustrate successful implementations of AI-powered phenotyping and breeding frameworks for major traits across diverse crop species. Furthermore, AI tools show great promise in refining crop traits at the molecular level by increasing the accuracy and precision of emerging fields including gene editing and genomic selection. We emphasize the importance of interdisciplinary collaboration to maximize the benefits of AI in plant breeding programs and to support the sustainable and food-secure future. This review bridges the gap between AI and agricultural applications, offering a roadmap for researchers, industry professionals, and policymakers to harness information fusion and computational models for advancing precision agriculture. It will serve as a valuable resource for future plant breeding, accelerating crop improvement from phenotyping to genomic selection and breeding decision support.

**Supplementary Information:**

The online version contains supplementary material available at 10.1007/s00122-026-05293-8.

## Introduction

Since the beginning of human civilization, people have consistently sought practical solutions to fulfil their most fundamental need: food. However, modern agriculture—the cornerstone of global food security—is currently facing three major concurrent challenges: climate change, resource depletion, and rapid population growth (Shebanina et al. 2024; Islam 2025). Although the precise impacts of climate change are difficult to predict, consensus suggests a generally negative effect on global food productivity (Hu et al. 2024). These adverse effects are driven by elevated atmospheric CO₂ levels and rising global temperatures (Vanaja et al. 2024; Boretti 2025), which have increased at an average rate of approximately 0.27 °C per decade in recent decades (Forster et al. 2024), together with increased pressure from pests and diseases (Ali et al. 2024a) and reductions in grain yield, milling quality, and other agronomic traits (Verma et al. 2025). Furthermore, climate change is associated with more frequent and severe extreme weather events, such as droughts and floods. To address these challenges, plant breeding has emerged as a crucial technological pillar of sustainable agriculture. Over the past few decades, plant breeding has made tremendous progress and has evolved through successive technological stages, commonly described as Breeding 1.0 to Breeding 5.0 (Kuriakose et al. 2020; Yoosefzadeh-Najafabadi et al. 2023; Fang 2024; Sangjan et al. 2025; Fu et al. 2025). Breeding 1.0, dating back 10,000–12,000 years, was based purely on phenotypic selection, whereas Breeding 2.0 incorporated foundational genetic principles, Mendelian and quantitative genetics, a reliance on controlled crosses, statistical models, and breeder experience (Wallace et al. 2018). Breeding 3.0 uses molecular markers and genomic information to complement phenotypic data, and high-throughput genotyping has emerged as a valuable tool for analysing natural populations and the implementation of genomic selection (GS) strategies (Zhu et al. 2024). Breeding 4.0 has been characterized by the deeper integration of genomic resources, advanced computational tools, high-throughput phenotyping (HTP), and the broader adoption of plant transformation and gene-editing technologies within breeding pipelines (Ramstein et al. 2019; Kuriakose et al. 2020; Fang 2024). In this phase, machine learning (ML) approaches increasingly support predictive breeding and data interpretation, enabling the analysis of complex, high-dimensional datasets beyond traditional analytical capacity (Yoosefzadeh-Najafabadi et al. 2023; Priyadarshan and Ortiz 2025). More recently, several authors have proposed the concept of Breeding 5.0, which emphasizes a further shift towards hyper-intelligent, AI-centric breeding systems characterized by cross-disciplinary convergence, generative foundation models, and increasing automation (Kuriakose et al. 2020; Yoosefzadeh-Najafabadi et al. 2023; Fang 2024; Fu et al. 2025). This emerging paradigm is supported by four core technological pillars: high-dimensional and multimodal data integration, omni-simulated breeding environments, people-less management and data acquisition, and expert and explainable artificial intelligence (Fu et al. 2025).

Despite these advancements, most breeding programs globally, especially in developing countries, continue to operate primarily within the Breeding 2.0 framework. This disparity between available technological potential and actual implementation poses a challenge, and it is no longer sufficient to ensure global food security (Wallace et al. 2018). Against this backdrop, AI has emerged as a transformative technology across multiple domains, and its integration into the field of plant sciences has extraordinary potential to assist in breeding climate-resilient smart crops and sustainably managing plant life. AI is widely referred to as the simulation of human cognitive processes in machines programmed to mimic reasoning, learning, and problem-solving abilities (Gupta et al. 2024). In agriculture, AI empowers traditional practices by applying advanced models and algorithms to extract meaningful patterns from large-scale genetic, phenotypic, multi-omics, and environmental datasets. Modern AI systems are designed to integrate data from various sources, including Internet of Things devices, satellite imagery, and historical agronomic records (Misra et al. 2020). By continuously learning from new data, these algorithms adjust to evolving conditions on the farm, offering real-time insights that assist farmers in making informed decisions (Dhillon et al. 2023). Specific AI approaches, including ML models such as Random Forests (RFs), Extreme Gradient Boosting (XGBoost), Support Vector Machines (SVM), and Least Absolute Shrinkage and Selection Operator (LASSO) regression (Kang et al. 2020; Ali et al. 2024b; Khalilzadeh et al. 2024; Barpete et al. 2025), as well as deep learning (DL), a subset of ML, including architectures such as Long Short-Term Memory (LSTM) networks, Convolutional Neural Networks (CNNs), and Recurrent Neural Networks (RNNs) (Khaki and Wang 2019; Shook et al. 2021; Srivastava et al. 2022; Ali et al. 2024b), have been applied to integrate genomic, phenotypic, weather, and environmental data for predictive breeding, often achieving high predictive accuracy. These AI-based approaches facilitate the prediction of genotype performance and stress responses across diverse environmental conditions, thereby supporting data-driven breeding strategies for improved crop adaptation and resilience under variable growing conditions.

**Fig. 1:**
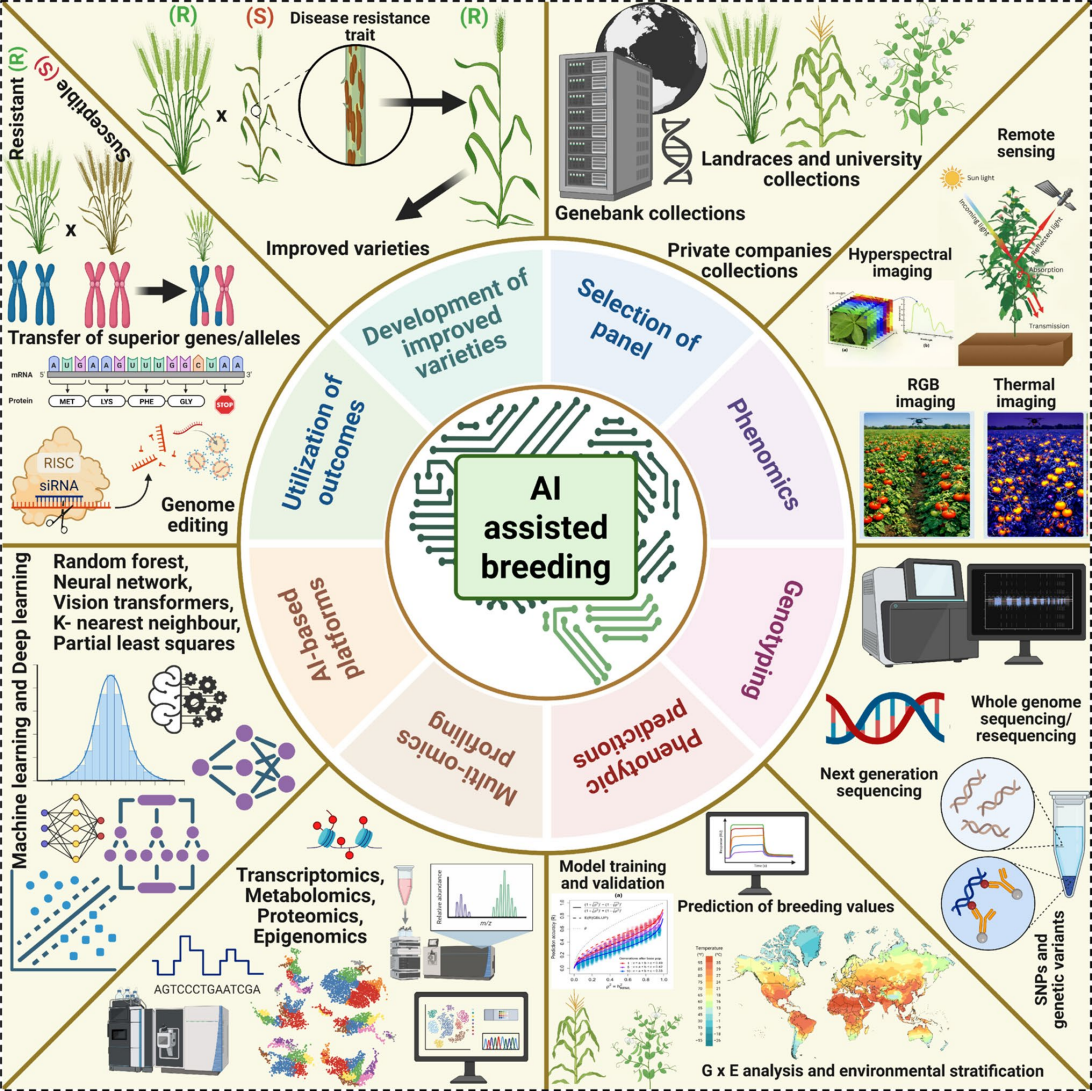
Strategic framework for AI-driven plant breeding innovation. This illustration presents an integrated, stepwise framework highlighting the role of AI across the plant breeding continuum. Selection of panel: initiates the process by choosing genetically diverse germplasm or breeding populations to capture maximum allelic variation. Phenomics: employs high-throughput phenotyping platforms (e.g. UAVs, sensor arrays, imaging systems) to collect accurate and dynamic trait data under diverse environments. Genotyping: involves sequencing or marker-based technologies to uncover genetic variants associated with traits of interest. Phenotypic prediction: integrates genotypic and phenotypic data using statistical models and machine learning algorithms to predict complex trait performance and breeding values across environments. Multi-omics profiling: combines genomics, transcriptomics, proteomics, metabolomics, and epigenomics to enhance biological insight and precision in trait dissection. AI-based platform: utilizes machine learning and deep learning algorithms for high-dimensional data analysis, feature extraction, pattern recognition, predictive modelling, and decision support. Utilization of outcomes: applies predictive outputs for marker-assisted selection, genomic selection, genome editing and identification of superior genotypes with enhanced stress resilience and productivity. Development of improved varieties: culminates in the release of improved cultivars with higher genetic gain, better adaptation, and resilience to climate and biotic/abiotic challenges (Har Fouche et al. 2019; Rai 2022; Farooq et al. 2024)

In the realm of plant phenotyping, AI-driven technologies facilitate automated, high-throughput analysis of complex traits and provide a deeper insight of plant growth and adaptation mechanisms for scientists (Khan 2025). These technologies assist in the identification of novel and potential gene–trait associations, the prediction of key agronomic traits, and the optimization of breeding pipelines. AI plays a central role in managing the massive datasets generated in phenomics, genomics, and environmental modelling (Khan et al. 2022). In parallel, genome editing technologies-including CRISPR–Cas9, zinc finger nucleases (ZFNs), and transcription activator-like effector nucleases (TALENs)-have revolutionized the field of molecular breeding by enabling precise and efficient genetic modifications (Ahmad et al. 2022; da Cunha et al. 2025). Although these tools offer distinct advantages, the design and optimization of editing strategies remain complex (Segelbacher et al. 2022). ML techniques are now increasingly utilized to analyse large-scale genomic data and predict off-target effects that increase the efficacy and specificity of genome editing systems (Pichler And Hartig 2023). A strategic integrative framework for AI-driven plant breeding innovation is illustrated in Fig. 1.

This review specifically focuses on recent advancements at the intersection of AI and plant breeding, focusing on (i) AI-integrated digital phenotyping; (ii) AI-enhanced breeding approaches—including GS, marker discovery, trait prediction, and gene editing; and (iii) the optimization of breeding pipelines through AI deployment. We also discuss ongoing challenges such as data heterogeneity, model interpretability, and the context dependency of AI models, and propose future research directions for fully utilizing AI vis-à-vis ML and DL in transforming crop improvement. AI is believed to be an indispensable tool for harnessing the benefits of these data technologies and reshaping the landscape of plant breeding.

## Intersection of AI and high-throughput phenotyping

### Revolutionizing phenotyping

In the field of plant science, plant phenotyping forms the cornerstone of any breeding program, as accurate measurement of plant traits is critical for selecting superior genotypes. Advances in next-generation sequencing have significantly accelerated progress in functional genomics (Bharathraj et al. 2025), enabling the widespread application of quantitative trait locus (QTL) mapping and genome-wide association studies (GWASs) (Thakur et al. 2024). These approaches have become powerful tools for dissecting the genetic architecture of complex traits, leading to the identification of numerous genes (Yang et al. 2024; Atsbeha et al. 2025; Kassie et al. 2025). However, the efficient acquisition of high-quality phenotypic data remains a major bottleneck, limiting progress in both crop improvement and functional genomics (Yang et al. 2020). Over the past decade, HTP has emerged as a transformative approach aimed at rapidly and precisely assessing phenotypic traits on a large scale. HTP integrates advanced technologies, automation, and data analytics to capture detailed measurements of various traits, such as plant growth, yield, stress tolerance, and disease resistance (Mir et al. 2019; Nguyen et al. 2025a). In contrast, traditional phenotyping methods are low throughput, labour intensive, time consuming, and prone to human error. HTP addresses these challenges by utilizing cutting-edge technologies such as remote sensing, advanced imaging systems, phenotyping platforms, and sensor networks to collect data from large numbers of plants simultaneously. Field-based phenotyping efforts have also been strengthened through mobile applications and integrated data platforms such as the FieldBook Android app, which enables efficient digital field data collection in experimental fields (Rife & Poland 2014), and BreedBase, a web-based breeding database system that supports breeding programs in managing and utilizing their data within a fully integrated digital ecosystem (Morales et al. 2022). These tools are especially beneficial for decentralized breeding programs operating across resource-limited environments, in which selection and evaluation are conducted across multiple target environments by geographically distributed teams, enabling explicit assessment of genotype × environment (G × E) interactions through breeding and testing directly within the environments where varieties will ultimately be grown.

AI is central to realizing the full potential of HTP. The integration of HTP tools with AI has significantly advanced crop stress phenotyping; however, many challenges remain unresolved, particularly when translating phenotyping approaches from controlled environments to open-field conditions. Controlled environment platforms (greenhouses and growth chambers) allow precise manipulation of abiotic factors (light, temperature, humidity, nutrients) and facilitate high-resolution phenotyping of specific traits under repeatable conditions. However, these conditions do not fully replicate the complex and heterogeneous environments of field conditions, where fluctuating solar radiation, wind, soil moisture, and biotic interactions strongly influence plant responses, often leading to divergent physiological and morphological outcomes (Morisse et al. 2022; Langstroff et al. 2022). Moreover, some HTP sensors or systems that perform robustly in greenhouse settings suffer reduced accuracy in open fields due to factors such as canopy shading, background interference, and soil heterogeneity (Gill et al. 2022). Consequently, accurate stress monitoring requires the integration of multimodal measurements that extend beyond imaging alone.

Modern HTP platforms therefore combine multiple imaging modalities and functional phenotyping approaches to quantify physiological and biochemical traits that are responsive to stress, including transpiration, stomatal conductance, water-use efficiency, and photosynthetic performance, enabling dynamic assessment of plant function under variable environmental conditions (Gosa et al. 2019; Li et al. 2021a; Gill et al. 2022). Automated systems such as PlantArray exemplify this approach by providing continuous, non-destructive measurements of whole-plant water relations and growth dynamics in response to water availability and biostimulant treatments (Dalal et al. 2019). At the molecular level, transcriptomic profiling captures stress‐responsive gene expression patterns that reflect specific environmental stimuli, providing mechanistic insights into how plants perceive and acclimate stress (Rurek And Smolibowski 2024). In parallel, chlorophyll fluorescence imaging enables phenotypic detection of stress-induced alterations in photosynthetic efficiency, further expanding the spectrum of observable stress indicators (Pérez-Bueno et al. 2019).

**Fig. 2:**
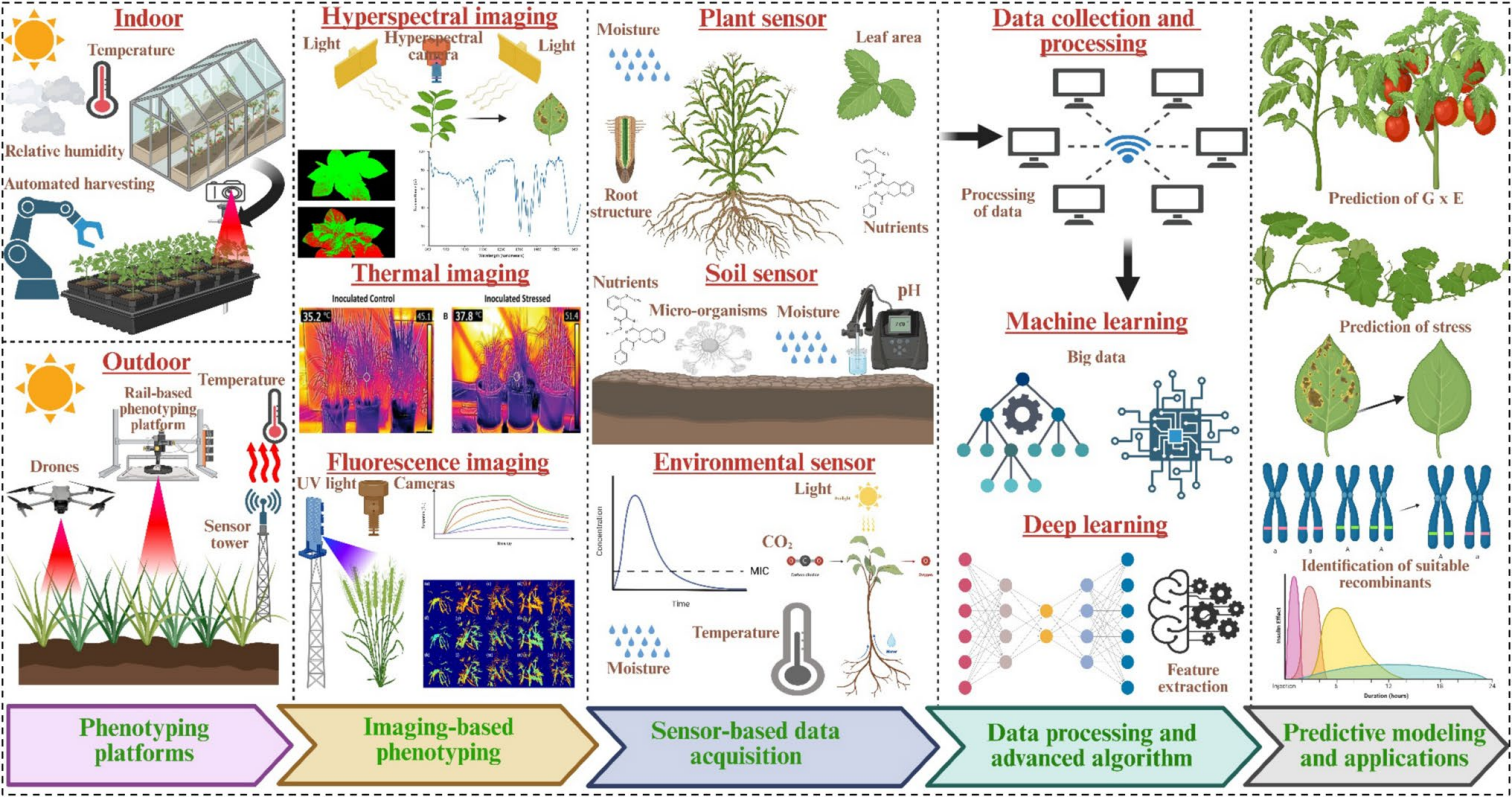
Conceptual framework for high-throughput phenotyping innovations. Phenotyping platforms: depicts diverse field-deployable phenotyping systems including unmanned aerial vehicles (UAVs), stationary towers, and rail-based platforms for dynamic and scalable data acquisition. Imaging-based phenotyping: highlights the integration of hyperspectral imaging for evaluating plant physiological status and biochemical composition; thermal imaging for assessing plant water relations and heat stress responses; and fluorescence imaging for monitoring photosynthetic efficiency and detecting early stress or disease symptoms. Sensor-based data acquisition: presents a range of sensor types employed in high-throughput phenotyping. Environmental sensors capture microclimatic variables such as light intensity, temperature, humidity, and CO₂ concentration. Soil sensors monitor key edaphic factors including pH and nutrient availability. Plant-based sensors assess morphological and physiological traits such as leaf area, growth dynamics, and chlorophyll content. Data processing and advanced algorithm: outlines the computational pipeline from raw data acquisition to phenotypic trait analysis. This includes data pre-processing, feature extraction, and the application of machine learning algorithms including deep learning-based models for pattern recognition and trait prediction. Predictive modelling and applications: illustrate the utilization of integrated genotype × environment (G × E) models to forecast phenotypic outcomes, facilitate the identification of stress-resilient genotypes, and inform the selection of optimal breeding recombinants (Farooq et al. 2024; Nguyen et al. 2025b; Cheng et al. 2025)

Critically, the accuracy, robustness, and transferability of AI-based phenotyping systems depend on the availability of high-quality and reliable ground-truth data, which are essential for model training, calibration, and independent validation (Henke et al. 2021; Tan et al. 2024; Varela et al. 2025). Multiple studies in automated plant image analysis consistently highlight inadequate or inconsistent ground-truth annotations as a major bottleneck limiting model performance and generalizability (Cruz et al. 2016; Henke et al. 2021; Ullah et al. 2025). To mitigate these limitations, approaches such as crowdsourced labelling and GAN-based data augmentation have been explored to improve training data quality (Zhou et al. 2018; Ullah et al. 2025). When supported by rigorously validated reference measurements across both controlled and field environments, AI-enhanced phenotyping frameworks can be deployed more reliably, enabling robust cross-environment comparisons of plant stress responses. A conceptual framework for HTP innovations is presented in Fig. 2.

### Technological foundations

Satellite imagery represents one of the earliest methods of HTP. It is capable of covering extensive areas ranging from 1000 hectares to entire counties at a time. Since the 1970s, satellite-based remote sensing has been employed to assess plant stress responses (Saini et al. 2022). A comprehensive list of unique satellite sensors and their spatial resolutions is detailed in Pettorelli (2019). More recently, the use of unmanned aerial vehicles (UAVs) for plant phenotyping and field crop monitoring has expanded considerably. UAVs, as components of unmanned aerial systems (UASs), include aerial platforms, ground-based controllers, various sensors, and communication systems (Chang et al. 2017). UAVs provide flexible, rapid data acquisition at high spatial resolutions (as fine as 1 mm/pixel) owing to their proximity to crop canopies. Compared with satellite images, UAV images have high resolution because they fly closer to the ground. Thus, remote sensing has been widely used for drought stress monitoring, nutrient status assessment, weed and pathogen detection, and yield prediction, as comprehensively reviewed in studies synthesizing progress in UAV-based remote sensing for crop phenotyping (Maes and Steppe 2019; Dhaliwal et al. 2024). Growing evidence also supports its application in genetic analyses, including QTL identification, as demonstrated across multiple studies (Hassan et al. 2021; Yan et al. 2023; Zhang et al. 2024a).

Canopy colour and texture traits captured by UAV platforms at high spatiotemporal resolutions facilitate phenotyping tasks since improvements in image quality and quantity provide detailed information for feature mining and analysis (Yue et al. 2019). Imaging via ground-based platforms is the most advanced technique. Owing to their close-range imaging capabilities, ground-based systems provide the most detailed phenotypic information (Bongomin et al. 2024). This proximity provides human-like manual phenotyping with improved efficiency. Typically, they can be equipped with onboard processors to automatically analyse phenotypic parameters in real time (Vougioukas 2019). These platforms are often equipped with a range of sensor devices, including RGB, multispectral (MS), hyperspectral, thermal, light detection and ranging (LiDAR), and fluorescence cameras. These sensors are essential for collecting the detailed data necessary for these platforms to perform effectively in agricultural tasks. However, the choice of which system and sensors to use depends on the research question, target species, and phenotypes of interest. RGB, or true-colour imagery, captures electromagnetic radiation at red, green, and blue wavelengths sensitive to light in the visible spectral range (400–700 nm) and is widely used to assess plant growth rates (Wasonga et al. 2021), classify plant images, measure plant height (Kior et al. 2024), analyse canopy coverage (Raman et al. 2022), detect disease (Tang et al. 2023), identify weed species, monitor crop senescence, and estimate biomass and yield (Liu et al. 2024a; Nikolova et al. 2025; Nakajima et al. 2025).

Multispectral (MS) sensors operate in a similar manner to RGB cameras but capture a broader spectral resolution and can be used to monitor spatial and temporal variations in vegetation indices (VIs). Several studies have validated the accuracy of VIs derived from aerially captured MS imagery for quantifying crop yield prediction (Maimaitijiang et al. 2020; Liu et al. 2023; Zhou et al. 2025), as well as for assessing plant health, moisture status, and nutrient content across diverse crops and environments (Babaeian et al. 2021; Sun et al. 2023; Gargiulo et al. 2024; Lazarević et al. 2024; Anzar et al. 2025). MS data have also been extensively applied for estimating chlorophyll content, leaf area index, and biomass (Gano et al. 2021; Guo et al. 2025).

Thermal imaging captures infrared radiation (9000–14,000 nm) in the electromagnetic spectrum and creates images based on it (Ramesh 2017). For plant phenotyping, thermography is particularly useful for assessing leaf surface temperature, which is correlated with transpiration rates and stomatal conductance (Mertens et al. 2023; Anderegg et al. 2024). Thermography has proven useful for the analysis of water and drought stress detection and is valuable not only for agricultural phenotyping but also for climate change research and vegetation analysis in non-crop and tree species (Lapidot et al. 2019; Seng et al. 2023; Yang et al. 2025a).

Hyperspectral imaging (HSI) captures electromagnetic spectra (*λ*) and spatial (*x*, *y*) data at every pixel in an image to reconstruct a 3D data matrix called a hypercube, which contains thousands of images in the spectral range of 250–2500 nm encompassing UV, VIS, NIR, and SWIR (Sarić et al. 2022). This technique has been widely exploited for nutrient estimation, stress detection (e.g. drought, heat, disease), fruit maturity assessment, and characterization of physiological and biochemical traits, which are used to infer plant growth and development as well as yield (Xie et al. 2024; Sherstneva et al. 2024; Ban et al. 2025; Rahman et al. 2025; Gámez et al. 2025; Wang et al. 2025a; Hu et al. 2025). LiDAR technology actively emits infrared laser pulses (typically 800–1000 nm), and the return speed and intensity are measured to determine the target height and reflectance properties (Chen et al. 2024). This active sensing method is particularly valuable for constructing 3D canopy models and quantifying traits such as biomass, canopy structure, and crop density across multiple cropping systems (Guo et al. 2018; Hütt et al. 2023; Panigrahi et al. 2025). Fluorescence imaging is employed to estimate photosynthetic efficiency and metabolic status under biotic and abiotic stress conditions (Kumar et al. 2021; Park et al. 2024; Zandi et al. 2025). Moreover, X-ray computed tomography (X-ray CT) can be used to visualize the 3D structures of the internal and external features of a plant at the micro- or macrolevel. This technique has been used to observe root growth (Kaur et al. 2018; Teramoto and Uga 2025) and characterize the size and shape-related morphological traits of seeds and fruits (Liu et al. 2020; Ahmed et al. 2020).

Multiple HTP platforms have been developed and are currently employed to phenotype a wide range of biotic and abiotic stress-related traits across diverse crop species. Details of selected imaging platforms used for trait phenotyping against various stresses were discussed by Gill et al. (2022) up to the year 2020. Additional platforms developed from 2020 to 2025 are presented in Table 1. These platforms are instrumental in deepening our understanding of stress adaptation mechanisms. The selection of a specific platform depends on the target trait, crop species, and experimental environment. For example, the CropDesign platform (Overijse, Belgium) was among the first controlled environmen, HTP systems to employ RGB imaging for crop analysis.

**Table 1:** Details of some recent platforms used for high-throughput phenotyping of morphological traits and assessing responses to biotic and abiotic stresses in crops

S.no	Crop	Platform	Traits recorded	Reference
1	Rice	PlantScreen™ robotic XYZ system	Drought	Kim et al. 2020
2	Horticultural crops	PHENOTIC	Identification of resistance to pathogens and virulence of pathogens	Boureau 2020
3	Cotton	PhenoRoots	Root related traits	Martins et al. 2019
4	Wheat	Self-propelled electric HTPP platform	Plant height	Pérez-Ruiz et al. 2020
5	Maize	MVS-Pheno	Plant height and leaf related traits	Wu et al. 2020
6	Cereals and Pulses	ChronoRoot	Root related traits	Gaggion et al. 2021
7	Sweet Pepper, Tomato	PATHoBot	Agro-morphological traits	Smitt et al. 2021
8	Wheat, Sorghum	PhenoImage	Plant responses to water stress	Zhu et al. 2021
9	Maize	EARBOX	Maize ears	Oury et al. 2021
10	Cereals and Pulses	RhizoPot	Root related traits	Zhao et al. 2022
11	Wheat	CropQuant-Air	Yield and yield-related traits	Chen et al. 2024
12	Arabidopsis thaliana	ScAnalyzer	Plant disease symptoms and pathogen spread	Paauw et al. 2024
13	Orchid	Pheno-Robot	Agro-morphological traits	Pan et al. 2024
14	Arabidopsis thaliana, Cowpea, Tepary bean	PhenoRig	Drought	Yu et al. 2024
15	Cereals and Pulses	ChronoRoot 2.0	2D-AI-Root related traits	Gaggion et al. 2025
16	Pepper	PhenAI-Bot	3D crop phenotyping (plant height, canopy major and minor diameters, canopy area, and leaf count)	Roy et al. 2025a
17	Pepper	Harvest-Bot	Precision harvesting of pepper	Roy et al. 2025b

The platform uses an automated conveyor-based plant handling system in a greenhouse, where potted plants (initially developed for rice) are transported to fixed imaging stations to quantify morphometric traits such as biomass, plant architecture, and colour. These measurements have been used to evaluate the effects of gene variants on agronomically important and yield-related traits in cereals (Reuzeau et al. 2010).

Yang et al. (2014) developed a high-throughput rice phenotyping platform using RGB imaging and linear X-ray CT to quantify 15 traits relevant to gene discovery. Controlled environment platforms such as PHENOPSIS and WIWAM are designed for precise trait dissection under strictly regulated conditions (Kuromori et al. 2022). Another key example is the PhenoArch platform (http://bioweb.supagro.inra.fr/phenoarch), hosted at the Montpellier Plant Phenotyping Platforms (M3P), a conveyor-based greenhouse phenotyping system designed for precise measurement of plant architecture, leaf area, biomass, individual organ size, and transpiration-related parameters. The platform enables the analysis of genetic determinants underlying plant responses to environmental conditions, particularly drought, temperature, and light (Cabrera‐Bosquet et al. 2016; Coindre et al. 2024). Commercial platforms such as LemnaTec, Phenovator, and Scanalyzer HTS offer modular solutions that combine RGB, infrared, near-infrared, and HSI to capture a wide array of phenotypic traits in controlled environments (Priya et al. 2025). For root system analysis, platforms such as GROWSCREEN-Rhizo and X-ray CT enable non-destructive analysis of root architecture, which is critical for understanding root-mediated stress adaptation mechanisms (Lube et al. 2022). Field-based phenotyping systems, including the Field Scanalyzer and TERRA-REF, integrate various imaging technologies with environmental sensors on gantry or mobile robotic frameworks (Gou et al. 2024). Together, these systems facilitate comprehensive, high-resolution field phenotyping, bridging the gap between genotype and phenotype by generating multidimensional, integrable datasets for genomic analysis.

### AI in data interpretation

For image analysis tasks that do not require complex pattern recognition, tools such as PlantCV, an open-source modular software suite for plant phenotyping, can efficiently extract biologically relevant measurements from images (Gehan et al. 2017). However, many modern image-based phenotyping platforms, particularly those operating at large scales and high temporal resolutions, generate massive datasets, often reaching terabytes per day, that cannot be efficiently handled manually or by simple statistical methods and therefore benefit from the application of AI-based approaches (Danilevicz et al. 2022; Gill et al. 2022). AI algorithms, including ML and DL, can process and integrate complex datasets, identify patterns, and make predictions to support decision making (Frimpong et al. 2025; Waqas et al. 2025). This is particularly advantageous in plant phenotyping, where AI can detect subtle variations associated with leaf colouration, texture, or disease symptoms that are associated with, or reflect underlying genetic traits.

AI models have been shown to predict agronomic traits such as crop yield and optimize crop management practices. In applied case studies, these models are implemented within data-driven decision support systems that integrate satellite and sensor-based remote sensing data, weather forecasts, soil information, crop health indicators, and historical farm records to generate timely and farm-specific recommendations. By analysing these multi-source datasets, such systems support yield forecasting, assessment of field and weather conditions, and provide targeted recommendations for irrigation, fertilization, and harvest scheduling, thereby reducing operational risks and improving resource-use efficiency. These predictive insights enable farmers to effectively plan their harvesting, storage, and marketing strategies (Karunathilake et al. 2023; Gupta and Kumar 2025). Since early innovations such as the BOPS-1960, a programmable unmanned tractor prototyped in 1960 at the University of Bologna, Italy, which employed early autonomous control mechanisms to execute field operations without human steering, AI research in agriculture has increasingly shifted towards ML-based approaches. ML models start by training on the dataset and are powered by algorithms such as SVMs, decision trees, RF, k-nearest neighbours (KNNs), logistic regression, clustering, dimensionality reduction, and artificial neural networks (ANNs) (Yoosefzadeh-Najafabadi et al. 2021; Shaikh et al. 2022). Most of the ML-based models require the accumulation of sufficiently large and high-quality data sets for their accurate and efficient outputs. Simulation-based studies have demonstrated that incomplete or low-quality training data significantly reduces model accuracy and robustness (Gong et al. 2023; Dube and Verster 2024; Mohammed et al. 2025). This challenge can be addressed through the integration of massive image datasets generated by HTP platforms.

ML techniques are broadly classified into supervised and unsupervised learning approaches, both of which have been extensively used in plant phenotyping for traits such as biotic and abiotic stress responses, biomass estimation, and yield prediction (van Dijk et al. 2021; Tatsumi et al. 2021; Zheng et al. 2022a; Gou et al. 2024). When data volumes become exceptionally large, DL, a subset of ML, becomes more suitable than traditional ML methods because of its scalability and ability to automatically learn complex representations (Khan et al. 2019; Ahmed et al. 2023). Unlike many conventional ML approaches, which require manual feature extraction prior to classification or regression, DL models can autonomously learn and extract features directly from raw data if the training data are well annotated (Paul et al. 2025). The most common algorithms used in DL are CNNs, RNNs, LSTMs, multilayer perceptrons (MLPs), generative adversarial networks (GANs), radial basis function networks (RBFNs) and vision transformers (ViTs) (Ahmed et al. 2023; Zhang et al. 2025).

DL models consist of multiple hidden layers in the network, each performing a specific operation to refine the input data progressively, thereby improving classification and prediction accuracy (Mienye et al. 2024). DL models are particularly well suited for analysing robotically captured images due to their ability to automatically learn hierarchical features directly from raw pixel data. These networks excel at tasks such as image recognition and segmentation, which are vital for robotic navigation and interaction with complex environments (Manakitsa et al. 2024), and have been successfully applied to automated root and seed feature recognition (Lube et al. 2022), leaf counting (Skoneczny et al. 2020) and plant stress identification and classification (Azimi et al. 2021; Goyal et al. 2024).

Despite the methodological differences, traditional ML approaches and their DL-based extensions provide excellent results when analysing HTP data. Therefore, selecting an appropriate AI technique based on the trait of interest, data characteristics, and platform constraints can increase the reliability and applicability of phenotypic predictions. Supplementary Table S1 summarizes case studies demonstrating the successful implementation of AI-powered phenotyping systems in the assessment of key traits across various crops. These studies are drawn from deep literature mining covering publications from 2006 to 2025 using targeted search terms such as “crop phenotyping”, “nutritional deficiency”, “biotic stress”, “abiotic stress”, “image analysis”, “hyperspectral or multispectral imaging”, “spectroscopy”, “machine learning”, and “neural networks”, along with crop- and trait-specific keywords.

## AI-driven breeding methodologies: advancing beyond traditional approaches

### Role of AI in genomic selection

To increase agricultural production and meet the expected rise in food demand in the coming years, plant breeding must yield the best rates of genetic gain. In this context, utilizing the potential of innovative approaches is the first step. One such innovative method is GS, a recognized breeding technique (Bernardo 1995; Meuwissen et al. 2001) that is based on predicting the breeding values of an unknown population by combining phenotypic data from an observed population with genome-wide DNA variation ("markers"). With the reduction in genotyping costs, GS has become a standard tool in many plant breeding programs, with the primarily application of shortening the length of breeding cycles and increasing the prediction/selection accuracy (González-Camacho et al. 2012; Crossa et al. 2013; Meuwissen et al. 2013; Krishnappa et al. 2021; Alemu et al. 2024). According to breeding studies conducted at the International Maize and Wheat Improvement Center (CIMMYT), GS can provide lines with noticeably improved agronomic performance while reducing the breeding cycle by up to 50% (Crossa et al. 2016). This reduction in cycle time is largely attributable to the application of GS in early breeding generations, including selection at the seedling stage, which enables earlier identification and advancement of superior genotypes. Genotypes with superior genomic estimated breeding values (GEBVs) can be rapidly recycled as parental lines, thereby substantially shortening the breeding cycle (Hickey et al. 2014; Bhat et al. 2016).

Over time, GS methods have developed markedly, moving from simple to more complex statistical frameworks. Originally, GWAS was designed mainly to test associations between individual markers and traits, typically using single‑locus models. Today, both GWAS and GS often rely on multi-locus, mixed-model or Bayesian approaches that can handle many markers and sometimes multiple traits simultaneously (Tibbs-Cortes et al. 2021; Lozano et al. 2023). GWAS aims to identify and interpret loci significantly associated with traits, whereas GS uses genome-wide marker information simultaneously to predict GEBVs without emphasizing individual marker effects (Zhang et al. 2023; Kumar et al. 2024). Meuwissen et al. (2001) made a significant contribution by introducing statistical models, including genomic best linear unbiased prediction (gBLUP) and several Bayesian techniques, that employed genome-wide marker data to more accurately predict GEBVs. This change made it possible for breeding programs to use selection more successfully, especially for complicated traits with several genes and small effects. When there were more markers than samples under study, researchers began employing a genomic relationship matrix, which is a summary of all marker data similarities across individuals, to improve prediction accuracy (Villanueva et al. 2005; Meuwissen et al. 2007; VanRaden et al. 2007; Verbyla et al. 2012). However, GS is not without challenges, as these models may become too slow to operate in complex breeding operations, particularly when numerous breeding lines and environments are involved (Norman et al. 2017). To address this, scientists have started utilizing fast computer methods, such as matrix algebra parallelization (De Coninck et al. 2015; Covarrubias-Pazaran 2016; Garrick et al. 2018), dimensionality reduction on the phenotypic space (Jighly et al. 2021), and ML (Azodi et al. 2019; Montesinos-López et al. 2021a), to increase the usefulness and efficiency of genomic prediction (GP). Further, to overcome the computational complexity in high-dimensional GP, newer hybrid AI models have been developed using deep kernel learning and ensemble methods, which combine biological priors with neural network flexibility to improve cross-environmental trait prediction (Cuevas et al. 2017). Such hybrid models outperform traditional gBLUPs, particularly when G × E interactions are strong (Crossa et al. 2019; Li et al. 2025a; Ye et al. 2025). Recent studies have demonstrated that the ensemble GP approaches integrating chromosome-partitioned marker information with Bayesian, BLUP, and ML models can significantly enhance the prediction accuracy for complex quantitative traits. For instance, in one of our own studies, a chromosome-partitioning-based integrative framework substantially increased GP accuracy for cold stress tolerance in wheat relative to conventional whole-genome prediction approaches (Meher et al. 2026). ML, especially DL, has become increasingly relevant in this context, owing to its ability to model complex, high-dimensional data and perform computationally intensive tasks in crop and livestock agriculture (Meshram et al. 2021) (Table 2). Many DL methods have been evaluated to determine whether they may increase prediction accuracy over standard linear models in the context of GP, with mixed outcomes depending on trait architecture, dataset size, and modelling strategy (Azodi et al. 2019; Montesinos-López et al. 2021b) (Table 2). In many ML-based GP studies, DL methods are commonly implemented using MLPs, a class of ANN capable of learning complex patterns between genotype and phenotype data (Montesinos-López et al. 2018, 2020, 2021b; González-Camacho et al. 2018; Sandhu et al. 2021a).

**Table 2:** Application of deep learning/machine learning models for genomic selection in crops

S. No	Crop	Deep Learning model	Response Variable	References
1	Strawberry and Blueberry	MLP and CNN	Average fruit weight, early marketable yield, total marketable weight, soluble solid content, percentage of culled fruit	Zingaretti et al. 2020
2	Wheat	MLP	Fusarium head blight, yield, harvest index, spike fertility, thousand-grain weight	Montesinos-López et al. 2020; Guo et al. 2020
3	Arabidopsis	MLP and CNN	Arabidopsis traits	Pook et al. 2020
4	Maize and Wheat	MLP	Leaf spot diseases, Grey Leaf Spot	Pérez-Rodríguez et al. 2020
5	Soyabean	LSTM-MLP	Yield	Shook et al. 2021
6	Corn, Soybean	CNN-RNN, Att-LSTM, MLP, LSTM Att, CropYieldNet	Yield	Khaki et al. 2020; Lin et al. 2020; Gangopadhyay et al. 2020
7	Wheat	MLP	Yield, protein content	Sandhu et al. 2021a
8	Maize	CNN, MLP, LSTM	Yield	Washburn et al. 2021; Sharma et al. 2022b
9	Barley	MLP	Yield	Maloy et al. 2021
10	Maize	DNN-CO and DNN-SO	Grain yield	Kick et al. 2023
11	Wheat	MLP and ResNet	Grain yield and thousand-grain weight	Montesinos-López et al.^2024^
12	Wheat	Multimodal PheGeMIL	Grain yield	Togninalli et al. 2023
13	Maize	PLS, RF, and GaussprRadial	Grain yield	Wu et al. 2024
14	Winter wheat	DNN	GY, TW, and GPC	Kaushal et al. 2024
15	Maize, Wheat, Foxtail millet, Rice, and Tomato	Cropformer	Quantitative traits	Wang et al. 2025b
16	Maize	AutoGP	Days to tasseling and plant height	Wu et al. 2025b
17	Wheat and Maize	MtCro	Agronomic traits	Chao et al. 2025
18	Cannabis	Machine learning algorithm	Cannabinoid compounds	Najafabadi and Torkamaneh 2025
19	Rice	RF and K-Nearest Neighbours	Yield and plant height	Bejjam and Basuthkar 2025
20	Eucalyptus	CNN and MLP	Tree height (TH), bifurcation height (BH), diameter at breast height (DBH), slenderness coefficient (SC), and volume (VOL)	Mora-Poblete et al. 2024
21	Grapevine	DCNN	Pest resistance	Gan et al. 2025

To handle high-dimensional genotypic data complexity, several DL–based GP approaches have been developed. Methods such as DNNGP (deep neural network for genomic prediction) (Wang et al. 2023) and DeepGS, an R package for predicting phenotypes from genotypes (Ma et al. 2018a), employ DL networks and strategies such as convolution layers, sampling, dropout, and ensemble learning to capture complex genotype–phenotype relationships (Ma et al. 2018a).

Beyond genotype-only prediction, DL models have increasingly been applied to integrate multi-omics data in plant breeding. DNNGP uses a multilayered hierarchical neural network structure to integrate multi-omics datasets, enabling dynamic feature learning and delivering comparable or superior prediction accuracy with reduced computational time relative to some conventional approaches (Wang et al. 2023). In addition, DeepInsight enables the transformation of non-image genomic and other omics data into structured representations suitable for convolutional neural network architectures, thereby facilitating genomic and multi-omics analyses using DL models (Sharma et al. 2019). Additional initiatives, such as NetGP, which combines genomic and transcriptomic data within a deep learning framework and achieves improved prediction performance across multiple plant species by capturing complex gene network interactions (Zhao et al. 2025). Multimodal DL approaches further enhance trait prediction by integrating genomic, phenomic, environmental, and management data, with architectures such as bidirectional long short-term memory (Bi-LSTM) networks and Transformers effectively modelling time-series environmental variables (Zou et al. 2025). Other advanced frameworks, such as MtCro, employ a multi-task learning strategy to jointly model multiple correlated plant phenotypes within a shared parameter space, resulting in improved prediction accuracy and model training efficiency (Chao et al. 2025).

Despite these advances, comparative studies indicate that DL models do not universally outperform the standard linear models (Montesinos-López et al. 2018, 2021a, 2021b). de los Campos et al. (2013) conducted a comprehensive comparison of whole-genome prediction (WGP) methods used in GS which evaluated both penalized and Bayesian regression approaches, encompassing parametric and nonparametric models, across simulated and real datasets. The analysis revealed that the majority of published studies relied on linear additive models, with GBLUP and Bayesian regression methods (e.g. BayesA, BayesB, BayesC, and Bayesian LASSO) being the most frequently applied and consistently performing approaches. Ma et al. (2018a) showed that DeepGS outperformed rrBLUP primarily when paired within linear models in an ensemble framework and suggested that both DL and rrBLUP models should be used for selecting the “best” individuals. Results from Sandhu et al. (2021a, 2021b, 2021c) indicate that DL approaches can provide modest gains in prediction accuracy over rrBLUP under specific conditions, often accompanied by increased computational cost. Similarly, Montesinos-López et al. (2018) compared the DL and gBLUP models across nine published genomic datasets (three maize and six wheat) and observed that DL performance outperformed gBLUP mainly when G × E interactions were ignored.

Methodological limitations of DL-based GP models have also been noted. In particular, DeepGS and DNNGP primarily rely on one-dimensional SNP vectors as model inputs, which can restrict their ability to effectively represent complex SNP locus features and capture the full extent of genotypic variation (Gao et al. 2023). Moreover, the shallow and wide convolutional architectures adopted in these approaches may be suboptimal for learning intricate relationships within high-dimensional genomic data.

To address these limitations, Gao et al. (2023) developed SoyDNGP, a deep learning–based GP framework that improves feature representation and prediction accuracy. Unlike DeepGS and DNNGP, SoyDNGP encodes genotypic data as a three-dimensional matrix, enabling more effective convolutional feature learning. The model uses a deeper, narrower convolutional architecture with stacked small kernels and stride-based downsampling instead of max pooling, reducing feature loss. Regularization strategies, including Dropout, Batch Normalization, and L2 penalties, are incorporated to enhance training stability, and a coordinate attention mechanism is used to jointly model spatial and channel information within the feature matrix, thereby strengthening feature extraction. Across multiple traits, datasets, and crop species, SoyDNGP consistently outperformed DeepGS and DNNGP while maintaining a modest increase in model complexity (Gao et al. 2023).

Additional DL architectures have also been proposed by Liu et al. (2019) to predict quantitative traits directly from SNP markers, in which missing genotypes are coded as an additional input category rather than being imputed. In this framework, a dual-stream CNN architecture was evaluated on both simulated and experimental soybean datasets, and demonstrated higher phenotype prediction accuracy in predicting multiple quantitative traits than several traditional statistical methods and simpler network architectures. More recently, a DL framework based on Elastic Net feature selection combined with Transformer-based embeddings and multi-head attention pooling (EBMGP) was introduced. By reducing input dimensionality through Elastic Net selection, EBMGP lowers computational burden while improving predictive performance, outperforming conventional one-hot encoding approaches and showing gains comparable to SoyDNGP (Ji et al. 2025).

However, although both EBMGP and SoyDNGP show promising performance, several aspects require further refinement. These models typically involve extensive parameter tuning, which can limit their usability, and their performance tends to be less stable on smaller datasets, indicating a need for improved feature selection and regularization strategies to reduce overfitting under data-limited conditions.

Overall, current evidence suggests that DL models offer valuable tools for GP and multi-omics integration, particularly for large and complex datasets, but their advantages over well-established linear models are context-dependent. In many scenarios, DL methods perform comparably to traditional approaches and may provide the greatest benefit when deployed alongside linear models within ensemble or hybrid frameworks rather than as universal replacements.

Furthermore, combining speed breeding with AI–enabled GS can accelerate breeding progress by substantially reducing generation time and increasing the rate at which genotypic and phenotypic data are generated. Speed breeding allows rapid population advancement and earlier allele fixation under controlled conditions, facilitating the development of larger training populations within a shorter timeframe for GP model development and updating. AI-based GS frameworks particularly benefit from this accelerated data generation, as model performance depends strongly on training population size and data density. However, phenotypes generated under speed breeding conditions may not fully reflect field-level G × E interactions, and therefore predictions derived from such data require validation under target production environments. When applied as a complementary strategy alongside multi-environment field trials, the combination of speed breeding and AI-driven GS provides a practical approach to increasing selection intensity and shortening breeding cycles without replacing field-based evaluation (Harfouche et al. 2019; Razzaq et al. 2021; Rai 2022; Bhat et al. 2023).

In addition, AI-based algorithms driven by OMICS data also help predict heritable changes, shorten breeding cycles, and improve yield (Xu et al. 2022). The efficiency of these AI-based algorithms is enhanced by using suitable neural networks and integrating them with computational models, making data interpretation more accurate and efficient (Sinha et al. 2023). Most current breeding initiatives are focused on creating climatic smart crops, by using GS models that leverage genome-wide marker information, including SNPs and InDels, for prediction of complex trait performance and breeding values (Kumar et al. 2024). Additionally, AI analyses the data produced by these models to accurately anticipate heritable components, reducing the breeding cycle and increasing plant production (Edukondalu et al. 2026). Thus, the integration of AI in crop breeding is a paradigm shift with important implications, offering improved selection accuracy, quicker breeding cycles, and adequate management of complex genomic data.

### Role of AI in gene editing

A major advancement in modern biotechnology is gene-editing technology, which enables researchers to precisely alter the genome at specific sites. Researchers can improve genetic traits and examine how genes function by using gene editing to add, remove, or replace specific regions in an organism’s DNA (Zhang et al. 2018; Jiang et al. 2022). As AI technology continues to advance, generative AI based on massive language models is quickly advancing genome editing technologies. With its ability to predict the effectiveness of various editing sites, intelligently optimize genome editing systems, and design different genome editing techniques based on target qualities, AI is actively transforming the area of genome editing (Li et al. 2025b; Thomson et al. 2025; Ruffolo et al. 2025; Jiang et al. 2025; Pandey et al. 2025). Although gene editing with CRISPR-Cas technology has advanced significantly, several obstacles remain to be overcome before it can be widely used. The two major challenges are editing efficiency and off-target consequences (Zhang et al. 2015a; Guo et al. 2023; Pena-Gutierrez et al. 2025). When guide RNA (gRNA) attaches to non-target DNA sequences, off-target cleavage events may occur, resulting in unintended genome modifications such as nonspecific double-strand breaks and sequence alterations. These off-target events can induce genomic instability, including insertions, deletions, and large structural variants, with potentially unpredictable molecular consequences (Höijer et al. 2022; Boutin et al. 2022; Tsai et al. 2023).

Furthermore, the efficacy of CRISPR–Cas editing can be improved, particularly in crops with numerous epistatic gene interactions and complex genomic backgrounds such as polyploidy and gene redundancy. AI technology has demonstrated significant promise in refining the CRISPR‒Cas system to overcome these issues. By using ML–based algorithms to estimate gRNA specificity and efficiency, AI-driven approaches enable the optimization of gRNA design to minimize off-target effects (Abadi et al. 2017; Zhong et al. 2023; Li et al. 2024a; Daneshpajouh et al. 2025; Yuan et al. 2025). These models leverage existing gRNA datasets to identify sequence features associated with high editing efficiency and specificity, thereby supporting more precise CRISPR–Cas genome editing.

The application potential of CRISPR-Cas9 in crop enhancement is greatly increased by this optimization approach (Chen et al. 2025a). In parallel, tools like AlphaFold, an AI system developed by DeepMind, are transforming protein structure prediction, which is fundamental not only for understanding CRISPR-Cas protein interactions and off-target effects, but also for predicting the functional consequences of sequence variants by revealing how amino acid changes may alter protein structure, stability, and molecular interactions. This structural insight supports the rational design of more efficient and specific genome editing systems (Jumper et al. 2021). The next section explores the distinct roles that AI plays in the field of genome editing.

AI-based methods have demonstrably outperformed traditional, mainly manual or simple statistical approaches for gene/trait target discovery in crops, both in accuracy and efficiency. Machine‑ and deep‑learning models can capture non‑linear, polygenic genotype–phenotype relationships and genotype‑by‑environment interactions that classical QTL mapping, GWAS and linear models often miss, leading to more accurate prediction of complex traits such as yield, drought tolerance and disease resistance (Farooq et al. 2023; Liu et al. 2024b; Alemu et al. 2024; Kassem et al. 2025; Gao et al. 2025). For example, in drought stress transcriptomics, integrating feature selection and random-forest–based ML increased classification accuracy from 0.889 to 0.942 and AUC from 0.968 to 0.978 for identifying key drought-responsive genes in wheat, highlighting quantitative gains over conventional differential-expression and co-expression analyses alone (He et al. 2025a). Similarly, the SaGP machine-learning pipeline for saline–alkali tolerance genes outperformed BLAST-based annotation and correctly predicted functions for newly published tolerance genes, enabling large-scale in-silico candidate discovery before costly wet-lab validation (Qiao et al. 2025). Across these studies, improvements are largest when models are trained on large, high-quality, well-curated multi-omics and phenotypic datasets, and when environmental data are integrated, whereas performance tends to plateau with small or noisy datasets, underlining that AI’s advantage depends critically on input data volume and quality (Wang et al. 2023; Wójcik-Gront et al. 2024; Alemu et al. 2024; Lell et al. 2025; Zou et al. 2025; Wu et al. 2025a).

Additionally, AI can simulate and forecast the results of various gene-editing techniques, assisting researchers in selecting the best course of action. AI can, for example, assess the specificity and cutting efficiency of several Cas variations and suggest the most appropriate editing tool for a given target. These techniques greatly increase the accuracy and effectiveness of gene editing, offering compelling evidence in favour of the wider use of gene-editing technologies (Jiang et al. 2025). Profluent, a company that designs AI proteins, announced the OpenCRISPR™ effort and released OpenCRISPR-1, the first open-source AI-generated gene editor in history (Ruffolo et al. 2025). Through extensive data mining, the study team created the most thorough CRISPR‒Cas Atlas (Madani et al. 2023) available. Using this dataset to train the ProGen2 model, approximately 4 million potential novel CRISPR‒Cas protein sequences were screened.

Automated gene-editing platforms are becoming a reality when paired with AI and liquid‑handling workstations and robotic microinjection or electroporation platforms that execute CRISPR or transfection protocols in multi‑well plates with minimal human intervention (Rigoulot et al. 2023, 2024). Liquid-handling robotic systems are increasingly being adopted in high-throughput plant genotyping workflows for automated DNA extraction and sample processing, enabling efficient handling of up to 384 plant samples per run while improving reproducibility, scalability, and minimizing cross-contamination risks compared to manual workflows (Boucher St-Amour et al. 2025). By facilitating high-throughput gene-editing experiments, these systems greatly improve the precision and efficiency of editing. Through real-time data analysis and feedback, AI plays a crucial role in these systems by streamlining the trial procedure and minimizing human error. This intelligent approach to gene editing not only increases efficiency but also improves experimental precision and reliability, providing essential support for the development of gene-editing technologies (Jiang et al. 2025).

Thus, genome editing technology should be integrated with complementary advancements, such as speed breeding and high-throughput phenomics, supported by AI-driven analytics and automation, to maximize their impact on agricultural productivity (Razzaq et al. 2021; Li et al. 2025c; Bradbury et al. 2025; Xie et al. 2025).

Although there are still regulatory barriers to the commercialization of genome-edited crops, an increasing number of nations exempt certain genome-edited crops (especially transgene-free SDN1/SDN2 edits) from strict GMO regulation, facilitating wider agricultural adoption (Menz et al. 2020; Turnbull et al. 2021; Tachikawa And Matsuo 2024; Hernández-Soto and Gatica-Arias, 2024; Fernández Ríos et al.^2025^; Lokya et al. 2025).

In addition to technological developments, a coherent pipeline involving cooperation between biotechnologists, agronomists, engineers, plant breeders, farmers, agribusinesses, and policymakers is necessary to achieve significant progress. To ensure that advances in genome editing and AI-driven technologies translate in to practical agricultural solutions that address global yield stagnation, food security, and climate resilience, there must be stronger collaboration between these sectors.

### The role of AI in marker-assisted selection

In crop breeding, marker-assisted selection (MAS) accelerates genetic gain by enabling the selection of individuals carrying favourable alleles using DNA markers, but its effectiveness depends critically on accurate phenotyping and robust marker–trait associations (Anand et al. 2023; Chang-Brahim et al. 2024). By combining ML with proximal or UAV-borne remote sensing, HTP can now quickly and non-destructively measure complex field traits like biomass, canopy temperature, stress responses, and yield components, overcoming major bottlenecks in traditional phenotyping (Ampatzidis And Partel 2019; Anand et al. 2023; Chang-Brahim et al. 2024; Kaushal et al. 2024). Crucially, HTP contributes to MAS or GS only when the sensor platforms reliably capture the target trait or a validated proxy, such as vegetation indices for biomass or canopy temperature for stress responses (Zhou et al. 2022; Chang-Brahim et al. 2024; Kaushal et al. 2024; García-Barrios et al. 2025). These sensor-derived phenotypes can then be incorporated into QTL mapping and GWAS, where AI/ML models have been shown to improve the detection of marker–trait associations and the prediction of complex traits compared with traditional statistical methods (Sandhu et al. 2022; Sheikh et al. 2024; Chang-Brahim et al. 2024; Kassem et al. 2025). Accordingly, AI-enabled HTP enhances MAS in two complementary ways: first, by generating large, high-quality phenotypic datasets for marker discovery and validation, and second, by supporting phenomic and multi-trait GP frameworks that integrate molecular markers with image-derived traits to improve selection for yield and stress tolerance (Sandhu et al. 2022; Kaushal et al. 2024; García-Barrios et al. 2025; Kaya 2025). Explainable AI (xAI) further aids practical implementation by improving model transparency and helping breeders interpret which spectral or physiological features underpin marker effects and selection decisions in practical MAS pipelines (Chang-Brahim et al. 2024; Fu et al. 2025).

AI is used to increase accuracy and efficiency in agricultural breeding and improvement programs. Developments in genomics-assisted breeding and high-throughput phenomics present a fantastic chance to develop plant cultivars that are stress tolerant. High-throughput data pertaining to phenotyping, genotyping, and engineering can be processed with the use of new tools and sophisticated computing facilities made possible by AI (Thudi et al. 2021; Zhao et al. 2022; Sheikh et al. 2024; Bhat et al. 2023; Cai et al.^2025^; Raza et al.^2025^; Angidi et al.^2025^; Sangjan et al.^2025^). AI technology makes it easier to identify and use genetic markers in breeding programs, and to link genotype to phenotype, particularly when combined with high‑throughput phenotyping and GP (Yang et al. 2020; Khan et al. 2022; Sheikh et al. 2024; Chang-Brahim et al. 2024; Kaushal et al. 2024; Li et al. 2024b; Thapa et al. 2024; Hussein et al. 2025).

AI-enabled marker-assisted breeding can improve the detection of selection sweeps for important agronomic traits by mitigating the effects of anomalous linkage disequilibrium (LD), which often arises from marker misplacement, genotyping errors, or population structure rather than true genetic linkage. LD-correction and related AI-enabled approaches use multi-marker LD patterns across the whole genome to re-assign such markers to positions where they show consistent LD with surrounding loci, thereby improving their physical alignment and reducing false positive signals in GWAS and sweep detection. This yields cleaner LD patterns that more reliably reflect true selection sweeps and agronomically relevant genomic regions (Dadshani et al. 2021; Yadav et al. 2021).

Grain yield and grain protein content in wheat can be predicted more accurately by optimizing multi-trait models via AI methods such as ML and DL. Furthermore, breeders can develop superior germplasm by using AI-based intelligent artificial climate chambers, which provide a location-independent and controlled environment for crop growth and phenotyping. In these systems, AI algorithms are integrated into environmental control modules to dynamically regulate growth conditions such as light, temperature, humidity, and CO₂, while computer-vision and machine-learning methods automate image-based trait extraction. This combination enables high-throughput, accurate, and non-destructive phenotyping under reproducible conditions, thereby supporting more efficient selection in breeding programs (Ren et al. 2022; ur Rehman et al. 2023; Kaya 2025; Chen et al. 2025b). Recently, xAI methods have been introduced into plant breeding workflows to open the ‘black box’ of ML models, improve trust, and extract biologically meaningful trait–genotype relationships (Mostafa et al. 2023; Novielli et al. 2024; Danilevicz et al. 2025). Overall, the state-of-the-art tools discussed above, including UAV-based phenotyping, GWAS, and MAS, supported by ML, along with the integration of emerging xAI, constitute a revolutionary change in plant breeding. These technologies collectively support the transition towards smart agriculture by enhancing data-driven decision-making and accelerating cultivar development (Xie and Yang 2020; Tanaka et al. 2024; Chang-Brahim et al. 2024; Mick et al. 2025).

## AI-assisted crop breeding

The intelligent and effective mining of big breeding datasets via appropriate models and robust algorithms is necessary for the implementation of next-generation AI in plant breeding (Harfouche et al. 2019). AI has emerged as a key driver for accelerating the process of crop improvement, as researchers have strived to develop and enhance its effectiveness to enable high-definition image recognition for processing complicated datasets (Harfouche et al. 2019; Godwin et al. 2019). AI approaches such as ANNs and DL are increasingly used to improve the efficiency and accuracy of multi-omics data analysis in plant science and breeding (Parmley et al. 2019). These models comprise multiple hierarchically organized layers of interconnected nodes that apply nonlinear transformations to input features, an architecture inspired by biological neural systems. While such designs enable powerful modelling of complex, high-dimensional biological data, the involvement of numerous hidden layers, nonlinear activation functions, and large numbers of trainable parameters obscures the direct relationship between input features and model outputs. As a result, the internal decision-making processes of ANN- and DL-based models are often difficult to interpret, giving rise to their characterization as “black box” systems (Rai 2022; Xu & Shuttleworth 2024).

In parallel, plant breeders are developing next-generation AI frameworks aimed at improving breeding value estimation and offering a thorough examination of complex traits in the face of shifting environmental conditions (Niazian and Niedbała, 2020). Additionally, repeated learning and improvement of AI will increase data mining efficiency and accuracy to better forecast the elements behind agronomic traits and disease resistance, thereby accelerating breeding efforts.

The development of high-throughput remote sensing and plant ‘omics’ techniques has given researchers access to vast multidimensional datasets for problem solving. The development of climate-resilient cultivars can be accelerated, and genetic diversity can be increased by the application of ML algorithms in adaptive marker-assisted selection, regional selection, and prebreeding techniques. The dynamic processes that lead to genetic variability, which are essential for plant adaptation to shifting climates, can also be preserved and restored by these algorithms (Yoosefzadeh-Najafabadi et al. 2024). Genetic gain is a result of the vast amount of biological diversity harboured within crops. More than 7 million germplasm accessions, including cultivars, landraces, and wild relatives, are conserved in approximately 1750 gene banks globally (McCouch et al. 2020), and their full potential is still unrealized. Genebank genomics, which involves genotyping stored germplasm resources genome wide, presents a promising way to better understand and make use of these valuable genetic resources. Notably, genome-wide genotyping data have been produced for a significant number of wheat, cacao, and barley accessions, with totals of approximately 80,000 (Juliana et al. 2019), 4000 (Romero Navarro et al. 2017), and 20,000 (Milner et al. 2019), respectively. AI-driven predictive genomics can use these datasets as a basis for the targeted selection and optimal testing of accession conditions.

It is quite difficult to obtain phenotypic information for such a large variety of germplasm resources under different environmental conditions. Environmental genome–wide association and genome–environment association (envGWAS/GEA) studies suggest that integrating genotypes with climatic and soil characteristics from georeferenced landraces can identify loci associated with local adaptation as shown in sorghum and other crops (Lasky et al. 2015; Ferrero-Serrano And Assmann 2019; Cortés et al. 2022). Studies such as those conducted by Lasky et al. (2015) have shown that the use of soil gradients and bioclimatic data from georeferenced sorghum landraces might reveal genetic markers linked to crop adaptability. Similar GEA-based methods have been presented for Arabidopsis, barley, and common beans to produce genome estimated adaptable values that may be utilized in GS frameworks and to prioritize accessions for stress adaptation (Ferrero-Serrano And Assmann 2019; Cortés et al. 2022; Halpin-McCormick et al. 2025). However, the applicability and robustness of these approaches may be limited since precise collection coordinates are frequently unavailable or inaccurate, and the substantial effort required to curate passport (basic descriptive and contextual information recorded for each germplasm accession at the time of collection and conservation) and environmental metadata. Furthermore, research on maize and wheat shows that, although environment-of-origin variables or envGWAS-prioritized SNPs can be useful in the absence of sequencing data, they usually only slightly improve predictive power for yield-related phenotypes beyond population structure and genome-wide markers (Xu et al. 2022; Li et al. 2024c, 2025d; He et al. 2025a, 2025b). Therefore, it is best to think of AI-driven GP models that incorporate dense markers, enviromic covariates, and curated passport data as complementary tools that can aid in identifying adaptive loci and ranking accessions for targeted environments, rather than as substitutes for conventional multi-environment phenotyping (Cortés et al. 2022; Xu et al. 2022; He et al. 2025b).

Several studies have shown how AI may be used to evaluate biochemical data and improve our knowledge of plant stress biology. To identify genomic areas with high mutation rates, for example, AI has been successfully applied to predict genomic crossings in maternal and parental maize plants (Farooq et al. 2024). Moreover, researchers have employed AI techniques to identify and define genomic regions on the basis of the DNA methylation patterns of maize plants developing under stress, thus distinguishing between functional genes and pseudogenes (Sartor et al. 2019). With the rapid advancement of sequencing technology, long-read sequencing has become the most widely used technique in crop breeding. Compared with short-read sequencing, this method has the advantages of allowing more structural variation and haplotype data to be obtained; nevertheless, it also presents more errors and difficulties in identifying variants (Watson And Warr 2019; Wenger et al. 2019; Conlin et al. 2022). Methods based on DL have been proposed to improve the accuracy and efficiency of variant calling from short-read sequencing (Poplin et al. 2018) and long-read sequencing (Ahsan et al. 2021; Zheng et al. 2022b), which are distinguished by their ability to extract significant patterns from complicated data. These methods outperform current tools and make it possible to find novel variants in difficult-to-map areas of the genome by utilizing a variety of techniques, such as image-based representation, local realignment, full-alignment, and haplotype-aware modelling, to take advantage of the complex information in long reads and reduce the impact of errors (Shafin et al. 2021).

The use of AI for accurate, non-destructive agricultural trait estimates and genetic research has gained popularity. Genetic advancements through the discovery of novel alleles and genomic-assisted choices in crop breeding are crucial to the development of cultivars with high yield potential that are adaptable to climate change. Numerous studies have effectively used AI-based techniques on RGB and HSI datasets to identify novel alleles in wheat, perform quantitative genomic analysis, and forecast early wheat production (Fei et al. 2022; Li et al. 2023). One of the main obstacles in genetic research is "missing heritability," referring to cases in which identified genetic variants fail to fully account for the heritable variation of complex traits (Manolio et al. 2009; Young 2019; Genin, 2020). This gap arises in part because complex traits are influenced by numerous rare variants, structural variations, and epistatic interactions that are not fully captured by standard GWAS approaches (Zuk et al. 2012; Young 2019; Carré et al. 2024). In these context, transposable elements and epigenetic alterations, such as DNA methylation, have been proposed as additional sources of heritable phenotypic variation that may contribute to explaining components of missing heritability (Trerotola et al. 2015; Bourrat and Lu 2017).

Inpactor2 (Orozco-Arias et al. 2023) and TEsorter (Zhang et al. 2022) are two examples of significant advancements in the identification and classification of transposons in plants via DL. AI-based approaches also show promise in supporting functional genomics interpretation and data-driven genomic design, thereby helping to bridge the genome–phenome gap (Telenti et al. 2018; Caudai et al. 2021; Hein et al. 2025).

Thus, the use of AI in precision breeding can improve crops faster and more accurately. This helps farmers grow better crops by selecting the best traits, such as higher yield or disease resistance.

## High-throughput breeding and automation

Agricultural productivity has grown dramatically over time because of intensification, mechanization, and automation (Nof 2009; Zhang 2013). This is a key aim for the use of many technologies intended to lower agricultural expenses while increasing crop output and quality. Precision fertigation, which involves applying water and plant nutrients to crops only at the appropriate time and location, lowers the ratio of agricultural inputs to crop production (Tremblay et al. 2011) and has a positive environmental impact (Tremblay et al. 2012). For example, optimizing in-season nitrogen application through remote sensing and fuzzy inference systems results in comparable yield as that obtained with the recommended uniform application, which requires 31% more nitrogen (Tremblay et al. 2010). More recent studies further demonstrate these benefits. In China, drip fertigation reduced evapotranspiration and allowed significant water and nitrogen savings without yield penalties, resulting in a 12% increase in crop yield, a 26% improvement in water productivity, and a 34% increase in nitrogen use efficiency (Li et al. 2021b). In soilless strawberry farming, sensor-based fertigation systems increased water-use efficiency by 46% and nutrient use efficiency by 74% while maintaining fruit quality (Bonelli et al. 2024). Similarly, improved precision fertilization combined with drip irrigation in Northeast China reduced nitrogen and phosphate inputs by up to 25% without adversely affecting maize growth or yield, thereby improving overall resource-use efficiency (Fan et al. 2020).

Furthermore, according to current research, using robots or autonomous tractors for a variety of agricultural operations reduces air pollution and fuel consumption (Gonzalez-de-Soto et al. 2016, 2015). The amount of labour that was historically available for farming activities was reduced by half throughout the twentieth century due to technological advancements in developed nations (Ceres et al. 1998). By improving efficiency, precision, and operational reliability while decreasing the need for continuous human intervention, automation has significantly increased the output of farming systems (Schueller 2006). However, despite the fact that farmworkers often possess significant crop-specific knowledge and practical expertise, agriculture—particularly the horticulture sector—continues to face labour shortages for physically demanding, repetitive, and time-sensitive tasks. In this context, robotics and automation can enhance agricultural output by reducing overall labour requirements and dependence on manual operations. Although these technologies require higher initial investments and specialized technical support, the reduced need for sustained manual labour and machine operation can offset these costs over time, resulting in net gains in productivity and operational efficiency.

Unlike the majority of robots in factories or cars in parking lots, agricultural settings necessitate that the robot be mobile (Canning et al. 2004). Autonomous robots frequently malfunction in these kinds of settings because of the numerous unforeseen circumstances (Steinfeld 2004). The requirement to operate in unstructured environments makes robotic applications more complex and significantly increases system cost. Robots are intelligent devices that may be trained to carry out particular jobs, make choices, and respond instantly. They are needed in a variety of industries that often require workforce and workload reductions, and they operate best in applications that demand high output and repeatable accuracy in stable environments (Holland and Nof 2007). However, they are unable to react to circumstances that are vague, unfamiliar, dynamic, and unpredictable. Two significant obstacles are commonly encountered in the design of autonomous robotic systems. The first focuses on the nonlinear, real-time response needs of the sensor control formulations. The second focuses on modelling and applying the human approach to each unique circumstance (Ng and Trivedi 1998). Beginning in the early 1960s, research on autonomous vehicles in agriculture focused mostly on the creation of automated steering systems (Wilson 2000). Heavy, strong, high-capacity machines that require considerable energy and have significant handling and operating expenses accounted for most mechanical operations in field crop farming in the 1990s. However, during the last two decades, research at numerous universities and research facilities worldwide has completely changed its paradigm from traditional heavy machinery towards more flexible, energy‑efficient robotic systems (Bechar And Vigneault 2016; Lowenberg-DeBoer et al. 2020; Fountas et al. 2020; Yang et al. 2023a).

## Optimizing breeding pipelines with AI integration

### Multidimensional data integration

The integration of AI into plant breeding methodologies signifies a fundamental transformation in the strategies used by breeders for navigating intricate biological systems and decision-making frameworks (Negus et al. 2024). Compared with the limited tools available previously, AI methods have become more practical in recent years, providing superior capabilities for managing and analysing large-scale, high-dimensional and diverse datasets ranging from genomic sequences, phenotypic traits, environmental parameters and management practices (Xu et al. 2022). Recent advances have demonstrated that the combination of multi-omics approaches, such as genomic, transcriptomic, metabolomic, phenomic, and enviromic information with AI is increasingly influencing predictive breeding strategies in plant science. For example, the integration of multiple omics datasets using a RF ML approach has been shown to improve prediction of complex agronomic traits, such as grain yield, relative to single-omics models (Wu et al. 2024**).** Similarly, ML models integrating multi-omics data have been applied to investigate molecular mechanisms underlying disease resistance and to support trait prediction in breeding programs (Mohamedikbal et al. 2025). Integrated multi-omics and AI frameworks have also been proposed for advanced plant phenotyping and precision agriculture, enabling the analysis of high-dimensional omics data in combination with environmental and management information (Cembrowska-Lech et al. 2023), and for climate-resilient crop breeding, facilitating improved trait predictability across diverse agroecological conditions (Amin et al. 2025). In parallel with these, AI-driven platforms can analyse massive, complex datasets to unravel interactions among genotypes, the environment, and management practices (G × E × M), enabling breeders to better understand how genetic potential is expressed under varying agroecological conditions (Zhang et al. 2024b). By recognizing hidden patterns and nonlinear interactions within these datasets, AI enables breeders to forecast how different genotypes respond to specific environmental and agronomic contexts, ultimately increasing the precision, efficiency, and resource optimization of breeding programs (Xu et al. 2022). A particularly promising application is the use of transfer learning to apply trained models across species or environments. Mahood et al. (2020) demonstrated that using CNNs pre-trained on one crop can enhance performance in distantly related species when adjusted with minimal data inputs, thus reducing model development time and improving scalability in low-resource breeding programs.

AI methods have proven especially valuable for addressing challenges such as data heterogeneity and cross-species knowledge transfer (Mahood et al. 2020). For example, CNNs integrated with HSI have improved wheat fusarium head blight resistance predictions by fusing phenotypic and genomic data (Thapa et al. 2024). Similarly, transfer learning has enabled disease severity classification across distantly related species such as cassava, strawberry, and grape, leveraging shared feature representations despite their genetic divergence (Yan et al. 2023). Recent case studies have also highlighted how models such as XGBoost and RF, which were initially trained on maize and Arabidopsis data, have been adapted to predict traits in rice, demonstrating the adaptability and scalability of AI-driven data integration across different crops (Cheng et al. 2021). Additionally, the combination of drone-derived imagery with CNNs and LSTM networks has optimized soybean maturity predictions, reducing the need for manual field assessments while maintaining high accuracy (Moeinizade et al. 2022). Consistently, Kim et al. (2025) developed a ML–based approach for soybean maturity prediction using time-series UAV RGB imagery transformed into contour plot representation, in which a DL model predicted relative maturity ratings and achieved up to 85% accuracy.

These examples collectively illustrate how AI harmonizes multiscale data, from molecular profiles to field-level environmental factors, to drive informed breeding decisions and resource-use efficiency. The ongoing development and application of these AI-based approaches signal a new era of precision, sustainability, and innovation in plant breeding (Sangjan et al. 2025).

### Predictive modelling for crop performance

The ability of AI to predict crop performance is transforming how breeders forecast crop performance. Leveraging ML and DL algorithms, such as regression models, decision trees, RFs, SVMs and DNNs, these models can simulate plant growth and yield under a range of climatic and management scenarios (Shingade And Mudhalwadkar 2024). This capability is particularly valuable in an era of increasing climate volatility, where traditional genotype evaluation may not sufficiently capture future environmental risks (Gerakari et al. 2025). Simulation tools powered by AI can model stress responses such as drought, salinity, or heat tolerance, helping breeders prioritize genotypes that are likely to thrive under projected environmental stresses (Raza et al. 2025). These models integrate historical yield data, weather patterns, soil characteristics, and management practices to simulate outcomes and identify varieties best suited for future challenges, leading to improved trait predictions and more efficient breeding cycles (Rai 2022). These models not only increase the accuracy of predicting traits such as yield, disease resistance, and maturity but also enable real-time or near real-time monitoring and decision-making, which is critical for within-season management and resource optimization (Banerjee et al. 2024; Shingade And Mudhalwadkar 2024). Moreover, these predictive frameworks can guide the spatial deployment of cultivars, aligning genetic potential with the environmental niches where they are most likely to succeed, supporting proactive breeding for resilience and yield stability (Raza et al. 2025).

For example, a study by Arumugam (2017) demonstrated the use of data mining techniques to predict paddy crop productivity via real-world data from farmers in South India. This study applied K-means clustering and various decision tree classifiers (including RF) to meteorological and agronomic data. The RF classifier achieved the highest prediction accuracy, and integrated clustering helped remove outliers, further improving the results. The research identified key trait combinations for high yield and generated actionable cultivation rules, enabling farmers to make informed decisions before harvest. In another study by Li et al. (2019), a statistical model was developed to predict rainfed corn yield in the U.S. Midwest via climate data (vapour pressure deficit and precipitation) and satellite-derived VIs. Their best-performing model, which combined climate variables with the enhanced vegetation index (EVI), achieved high predictive accuracy (*R*^2^ = 0.85, RMSE = 0.90 t/ha). The study revealed that integrating satellite data significantly improved yield prediction and highlighted the importance of model transparency and open data practices. Shahhosseini et al. (2021) demonstrated that coupling crop simulation modelling (APSIM) with ML methods (including RF, XGBoost, and LightGBM) significantly improved corn yield prediction in the US Corn Belt. Integrating crop model outputs—especially soil moisture and drought stress variables—with machine learning reduced prediction errors by 7–20%. The study revealed that hybrid approaches outperformed models that use weather data alone, highlighting the importance of hydrological and physiological variables for accurate yield forecasting. Collectively, these studies illustrate how predictive modelling, powered by advanced algorithms and multidimensional data, is revolutionizing crop performance forecasting and enabling more resilient, efficient, and sustainable agricultural systems.

Another active area is the simultaneous integration of mechanistic biophysical modelling with genomic prediction (CGM-WGP), which represents a significant advancement in GP by integrating the crop growth models (CGMs). GP generally works well in predicting the performance of unobserved individuals that are related to the reference population, but struggle to extend the prediction to unobserved environments. CGMs, on the other hand, simulate plant growth and development based on environmental and management inputs and genotype-specific parameters (GSPs), providing a biological framework to understand how genetic variations translate into phenotypes, which can facilitate the extension of prediction for existing genotypes to unobserved environments. The CGM-WGP approach, first published by Technow et al. (2015), aims to overcome the limitations of each standalone method to facilitate the prediction of new individuals in new environments. The model predicts the GSPs using genomic information, the CGM-WGP model can then simulate the performance of new genotypes in various environments through the CGM, including those not previously observed, thus capturing complex G × E × M interactions and offering more robust predictions for unphenotyped traits and future climate scenarios. The model has been successfully applied to maize (Technow et al. 2015; Messina et al. 2018), rice (Onogi et al. 2016), and wheat (Jighly et al. 2023a, 2023b, 2024). Further integration of this model with various AI-based strategies described here would boost its advantages and significantly improve the prediction accuracy of different traits.

### Decision support systems

To translate complex data into actionable breeding strategies, AI-powered decision support systems (DSSs) have emerged as essential tools in modern breeding pipelines. These systems process and analyse complex datasets generated by modern genomics and phenomics technologies and assist breeders in selecting parents, designing crosses, and allocating field trials by providing real-time, evidence-based recommendations (Mishra And Mishra 2024; Soori et al. 2024). Traditional breeding methods face bottlenecks in data management and analysis, but AI, particularly ML and DL algorithms, can extract meaningful patterns from high-throughput sequencing and imaging data, leading to less biased and more actionable insights (Harfouche et al. 2019; Zhang et al. 2024b). Unlike static decision-making matrices, AI-enabled DSS tools continuously adapt to incoming data from trials, remote sensing platforms, and laboratory assays (Yingngam et al. 2024). This dynamism allows breeding programs to pivot quickly in response to emerging challenges or promising discoveries, thereby accelerating genetic gain (Khalifa et al. 2024). For example, AI-driven GP models that incorporate georeferenced passport data and agroclimatic variables can help breeders select the best accessions even when traditional phenotypic data are scarce (Farooq et al. 2024). Tools such as large language models (LLMs) are also being explored for their potential to assist in data interpretation and generate intelligent queries relevant to plant breeding challenges (Feng et al. 2024; Shaikh et al. 2024). DSS platforms reduce uncertainty and facilitate risk assessment by evaluating the probability of success for specific breeding decisions, thereby improving the overall efficiency of breeding pipelines (Zhao et al. 2022).

HTP platforms exemplify critical data sources for DSS integration. The Rothamsted Research Field Scanalyzer and similar platforms such as the Crop3D HTP system, are automated systems that use advanced sensors, including RGB, thermal, hyperspectral, and 3D imaging, to measure crop canopy development, plant structure, and physiological traits at scale (Virlet et al. 2016; Sadeghi et al. 2017; Guo et al. 2018). While these platforms themselves do not constitute DSSs, the rich phenotypic datasets they generate can be integrated with AI-enabled DSS models to support informed breeding decisions, making them essential components within AI-driven predictive breeding frameworks. At the global level, several DSSs have been developed in the agricultural sector to forecast and recommend treatment programs against potato late blight via several disease forecasting systems and models (Soori et al. 2024). Among them, the most widely reported systems are SimCast, BliteCast, ProPhy, SimPhyt, Plant-Plus, MILEOS, Guntz-Divoux, and BlightPro (Krause et al. 1975; Nugteren 1997; Van der Waals et al. 2003; Kleinhenz & Jörg 1999; Grünwald et al. 2002; Michelante et al. 2003; Gaucher et al. 2014; Small et al. 2015).

## Global perspective on AI-driven breeding

AI is rapidly emerging as a transformative force in plant breeding worldwide (Sangjan et al. 2025) and is widely recognized for its ability to accelerate genetic gain and enhance agricultural sustainability in response to climate change and population growth (Rai 2022). The field is now characterized by a “data deluge,” with advances in genomics and phenomics generating unprecedented volumes of information that traditional methods struggle to manage (Koh et al. 2024). AI overcomes this challenge by enabling integrated data infrastructures and robust predictive models, which can process information from more than 7 million germplasm accessions stored in over 1750 gene banks around the world (Farooq et al. 2024). International initiatives such as the 10 KP plan within the Earth BioGenome Project involve the sequencing of 10,000 plant species to provide comprehensive reference genomes, making it easier to discover beneficial traits and transfer them into elite crop varieties (Gupta 2022). AI-driven breeding is being adopted across diverse regions to optimize crop rotation, intercropping, and adaptive marker-assisted selection, thereby significantly improving both resilience and productivity (Ansari et al. 2024; Kundu 2024).

The power of AI extends beyond data processing—it is fostering a new era of collaboration among researchers, breeders, and data scientists to address urgent global challenges such as food security, climate adaptation, and sustainable agricultural production (Pandey And Mishra 2024). Shared databases and AI-enabled platforms facilitate the exchange of germplasm and breeding information across borders, allowing for the identification of superior alleles and effective breeding strategies tailored to various agroecological zones (Ahmad et al. 2024; Ansari et al. 2024). Initiatives such as the CGIAR’s Excellence in Breeding (EiB) platform and other international breeding networks leverage AI to harmonize data collection, standardize phenotyping protocols, and optimize breeding workflows (McHugh et al. 2022). This collaborative approach is especially vital for developing and disseminating climate-resilient, high-yielding crop varieties that are adapted to the needs of smallholder farmers.

By making advanced tools and insights more accessible, AI is democratizing the benefits of modern breeding and ensuring that progress is not limited to industrial agriculture (Kumar et al. 2025). Case studies, such as the use of AI to analyse bioclimatic and soil gradient data of sorghum landraces by Lasky et al. (2015), highlight how AI can uncover genomic signatures of adaptability and guide breeding strategies even in data-limited contexts. Advanced phenotyping platforms such as the Rothamsted Research Field Scanalyzer and Crop3D systems further demonstrate AI’s role in monitoring crop canopy development and quantifying plant traits at scale, supporting large-scale breeding programs worldwide (Farooq et al. 2024). In essence, AI is reshaping the structure and function of modern breeding pipelines from data integration and trait prediction to strategic decision-making and global collaboration (Xu et al. 2022), providing breeders with the tools needed to address the urgent challenges of the twenty-first century (Zhang et al. 2024b).

## Challenges of AI in plant breeding and future directions

While the application of AI in plant breeding has significantly increased our ability to analyse and predict plant traits, several challenges and limitations remain. These issues can affect the accuracy, reliability, and scalability of AI-driven research and applications. This section highlights the limitations of AI in plant breeding and explores the most promising technologies and directions that researchers can consider for further investigations.

The cost of HTP data is a major limitation, making it unaffordable for many farmers, especially those from developing countries. This includes the expense of sensors, advanced robotics, and sophisticated data analysis systems (Sharma et al. 2022a; Qazi et al. 2022). Overcoming such challenges will require collaborative efforts from government bodies, technology companies, and academic institutions.

Another limiting factor is the storage, management, and processing of big datasets, along with the generation of valuable information at the biological level (Sheikh et al. 2024). Every year, thousands of phenotyping experiments carried out in controlled environments or field conditions generate vast volumes of data. However, replicating results within or across research groups are often not satisfactory due to the unexplained environmental variation (Sheikh et al. 2024). To manage and integrate such extensive datasets, Wilkinson et al. (2016) introduced the FAIR data principles (Findable, Accessible, Interoperable, and Reusable) to facilitate the finding and reuse of data across different users or groups, which means that all the necessary metadata, such as resource details and data acquisition information, measurement protocols, data descriptions, and environmental conditions, should be clearly addressed and capable of being accessed. Following these principles, several platforms have also been developed for data management and analysis, such as PHENOPSIS DB (Fabre et al. 2011) and CropSight (Reynolds et al. 2019), which have been developed to facilitate data management and analysis in accordance with these principles.

Data quality is another major concern and is affected by multiple factors, such as environmental variability, measurement inaccuracies, and human bias (Gano et al. 2023). For example, training AI models using an image dataset that consists of data points collected under highly controlled conditions (e.g. uniform lighting and temperature in growth chambers) can lead to biased predictions and fail to predict when the DL algorithm are applied to real-world images where conditions can vary significantly throughout the day/night cycle. Similarly, plants subjected to drought stress may exhibit different responses due to variations in transpiration rates. Even in apparently stable greenhouse conditions, measurements taken at different times may vary due to natural fluctuations in light intensity throughout the day (Mansoor et al. 2025).

Beyond data quality, context dependency represents a critical limitation in AI-based plant phenotyping and breeding, whereby model outputs are tightly coupled to the experimental context in which the data are generated. Tan et al. (2024) showed that DL models trained for plant trait extraction are highly sensitive to differences in species, imaging modalities, image quality, trait definitions, and annotation strategies, resulting in marked declines in performance when models are applied outside their original training context. The study demonstrated that models often capture context-specific visual patterns rather than biologically transferable trait representations, thereby limiting reproducibility and restricting reuse of trained models across experiments. Addressing this challenge requires diverse training datasets that represent a wide range of genetic, developmental, and environmental conditions, as well as the adoption of robust validation strategies that test model performance across multiple contexts. Transfer learning, domain adaptation techniques, and few-shot learning approaches offer potential solutions by enabling models to adjust to new contexts with limited additional data (Wu et al. 2023; Lagergren 2023; Tan et al. 2024). In many GP/GS trials, training data may be insufficient for capturing complex nonlinear interactions among a large number of markers. In such situations, linear models that focus on modelling additive effects may outperform nonlinear models (Bhat et al. 2023). Integrating high-dimensional phenomic data, including traits derived from high-throughput imaging and sensor platforms, into GP/GS models has been shown to enhance prediction accuracy by capturing aspects of plant performance and G × E interactions that are not fully captured by genomic markers alone (Adak et al. 2023a). However, biased, inconsistent or incomplete phenomic datasets pose challenges for training accurate and generalizable AI models, resulting in less reliable predictions and hindering the ability to apply models across different agricultural settings (Cembrowska-Lech et al. 2023). To address these data limitations, strategies such as crowdsourcing and community science have been proposed to generate larger and more diverse datasets (Palmer et al. 2021). Reviews on image analysis software and algorithms have been provided by Perez-Sanz et al. (2017) and Atkinson et al. (2019). A comprehensive understanding of plant traits requires acknowledging the dynamic interplay between plants and their environment (Naz And Afzal 2023). This raises the crucial question: how can we measure environmental impacts effectively? The environmental parameters must receive similar attention from researchers as the traits that need to be measured. Next-generation envirotyping technologies refer to high-throughput methods for systematically characterizing environmental factors that influence plant growth and yield, such as climate, soil properties, canopy conditions, crop management practices, and companion organisms, thereby providing data to address this issue (Xu 2016). The integration of multiple types of information, including G × E × M interactions, may usher in an era of predictive phenomics (Araus et al. 2018). Additionally, there is a need to build multidisciplinary groups that can address different challenges across the domains of biology, environmental sciences, and computer sciences.

Insufficient funding and inadequate data infrastructure also pose barriers (Moghayedi et al. 2024). A shortage of trained personnel, particularly data analysts and imaging specialists, further limits the widespread adoption of phenotyping tools (Tsaftaris And Scharr 2019). The global adoption of HTP platforms also carries risks (Zhao et al. 2019). For root phenotyping, methods such as the core-break technique for obtaining the depth of roots (Wasson et al. 2017) and minirhizotrons with sensors for observing the growth of roots (Svane et al. 2019) offer only partial insights, as they sample only a small portion of the root system. Non-destructive tools for detection, such as ground-penetrating radar (Delgado et al. 2017) and electrical impedance tomography (Corona-Lopez et al. 2019), offer broader detection of the biomass of roots and help capture the development of roots but suffer from low spatial resolution (~ 1 cm/pixel) and poor sensitivity to fine roots at the individual level. Spectrometers, which rely on solar radiation as a light source in field applications, are highly sensitive and face challenges such as cloud cover, shadows, and changes in the solar angle during the photo period, all of which impact image quality (Zhu et al. 2017). Another hurdle in HSI is redundancy arising from the continuous nature of wavelengths and their similarity. This is addressed via wavelength selection algorithms such as the successive projections algorithm (SPA), the genetic algorithm (GA), Monte Carlo uninformative variable elimination (MC-UVE), and boosted regression tree (BRT), which is also an ML technique (Gu et al. 2019). UAVs also face challenges, including data loss due to environmental factors, airspace restrictions, limited battery life and payload capacity, safety concerns, and interference from wildlife (Olson And Anderson 2021). Some companies, such as Harris Aerial, are developing UAVs with integrated electric or solar power systems. Technological advances—such as obstacle avoidance, GPS, accelerometers, gyroscopes, and advanced optical sensors—are improving UAV robustness under field conditions.

Another key challenge in applying AI to phenomics is interpretability, particularly with DL models. These often function as “black boxes,” making it difficult for researchers to understand how specific predictions are generated. This lack of transparency can hinder the widespread adoption of AI in plant science (Novielli et al. 2024). There is a need for more transparent and interpretable models that can be easily understood by plant biologists. There is a need for more generalized models that can be applied to a wide range of plant phenotypic datasets and species. To address the ‘black box’ problem of DL models, xAI tools such as SHAP (SHapley Additive exPlanations) and LIME (Local Interpretable Model-Agnostic Explanations) have been adapted for plant trait prediction pipelines. These allow breeders to interpret and trust AI decisions by identifying the most influential features behind each prediction (Molnar 2022).

The use of AI also raises ethical and regulatory concerns. One ethical concern arises with the use of AI in the manipulation of genetic material, which many say raises questions about the impacts that this could have in the longer term on biodiversity, food security, and the long-term ecosystem (Powers And Ganascia 2020). Other concerns include data privacy, ownership of phenotypic data, and the potential loss of traditional farming knowledge and practices (Tzachor et al. 2022). To address these concerns, robust regulatory frameworks must be established that balance ethical considerations with the need for innovation. By addressing these issues, agriculture can move towards a more productive and sustainable future, supported by AI (Sharma et al. 2023; Gryshova et al. 2024).

Another pressing challenge in plant science is the lack of transparency and trust in data sharing and collaboration (Kalita et al. 2026). Researchers often face difficulties in accessing or verifying datasets, which hinders progress and delays scientific discoveries. Open-source tools, devices, and blockchain-based platforms offer promising solutions by enabling secure, transparent, and verifiable data sharing (Jadav et al. 2023). One such tool is the MultispeQ device, a low-cost sensor linked to the PhotosynQ platform (https://www.photosynq.com), which supports community-driven, field-based phenotyping (Kuhlgert et al. 2016). Additional open-source initiatives such as Planteome (Cooper et al. 2018), BrAPI (Selby et al. 2019), Breedbase (Morales et al. 2022), AgTC and AgETL (Vargas-Rojas et al. 2024) contribute significantly to data sharing and collaboration in plant research and breeding.

## Conclusion

As the global agricultural landscape faces the compounded challenges of climate change, diminishing natural resources, and a rapidly growing population, it has become increasingly clear that traditional breeding approaches alone are no longer sufficient to meet future food security demands. This review highlights how the integration of AI, particularly ML and DL, into plant breeding pipelines is reshaping our capacity to address these issues with greater speed, accuracy, and scalability.

AI has demonstrated immense potential in accelerating the entire crop improvement process, from phenotyping and trait prediction to GS and breeding decision support. In particular, the synergy between AI and HTP has enabled unprecedented advancements in trait dissection, environmental interaction modelling, and non-invasive measurement of plant performance under both controlled and field conditions. Technologies such as UAVs, ground-based sensors, HSI, and LiDAR—when coupled with AI-driven analytics—facilitate dynamic, multidimensional data generation and interpretation. The integration of these tools with advanced AI-based predictive genetics tools as well as biophysical growth modelling would offer a more holistic understanding of G × E × M interactions, leading to the development of more resilient cultivars.

Furthermore, AI is revolutionizing molecular breeding by improving the precision of genome editing tools, predicting off-target effects, and guiding the *in-silico* design of functional variants. The deployment of interpretable AI models can support breeders in identifying novel alleles and creating climate-resilient cultivars with tailored agronomic traits. These developments are not merely theoretical; case studies and real-world applications across major crops have shown that AI-integrated approaches already outperform conventional methods in speed, accuracy, and predictive power.

However, despite this remarkable progress, several critical challenges must be addressed to ensure the sustainable and equitable implementation of AI in plant breeding. Data-related issues, including heterogeneity, inconsistency, and lack of standardization, continue to hinder model training and cross-platform interoperability. The high costs associated with HTP platforms and AI deployment restrict access in resource-limited settings, especially in developing countries where the need for resilient crop varieties is often greatest. Additionally, the black box nature of many DL models poses questions about interpretability, reproducibility, and user trust—particularly among domain experts who may not be versed in AI technologies.

The ethical, legal, and social implications of AI applications in agriculture also warrant careful consideration. Concerns related to genetic data privacy, algorithmic bias, loss of indigenous knowledge systems, and unequal access to technological innovations highlight the need for inclusive and transparent policy frameworks. Collaborative, interdisciplinary approaches that bring together plant biologists, data scientists, breeders, policymakers, and local stakeholders are essential for developing ethical AI systems that are not only technically sound but also socially responsible.

As AI tools become increasingly embedded within plant breeding pipelines, several emerging areas merit closer attention for their transformative potential. xAI has emerged as a critical component to enhance breeder trust in model outputs by enabling transparent interpretation of decision-making processes, especially in GP and trait selection (Holzinger et al. 2022). Simultaneously, the rise of pan genomics—which captures structural variants and rare alleles absent in reference genomes—has opened new avenues for AI-driven haplotype mining and trait association studies (Schreiber et al. 2024). In synthetic biology, AI accelerates DBTL (Design–Build–Test–Learn) cycles, supporting rapid metabolic pathway optimization, circuit design, and the development of synthetic phytobiomass tailored to specific agroecological contexts (Jervis et al. 2019). Furthermore, the integration of AI with climate modelling enables climate-smart GP, allowing breeders to forecast cultivar performance under future environmental scenarios and design region-specific deployment strategies (Tariqul Islam et al. 2026). Addressing model limitations such as overfitting, data imbalance, and generalization across genotypes and environments is also essential to ensure robust and reproducible applications (Montesinos-López et al. 2022). Incorporating these dimensions into AI-assisted breeding frameworks will foster scalable, ethical, and resilient agricultural systems for the future.

In conclusion, AI holds the transformative power to redefine plant breeding by unlocking deeper biological insights, enhancing selection accuracy, and enabling rapid development of superior cultivars tailored to specific agroecological contexts. The ability to identify hierarchical features and infer generalized trends from large datasets is one of the key attributes underlying the success of ML tools in plant breeding. The path forward demands a balance between technological innovation and practical deployment, ensuring that AI-enhanced breeding serves the broader goals of food security, environmental sustainability, and agricultural resilience. By fostering inclusive, collaborative ecosystems and addressing existing barriers, the plant science community can fully harness the promise of AI to usher in a new era of intelligent, data-driven crop improvement. Looking ahead, the convergence of AI with synthetic biology—especially through DBTL cycles—is poised to revolutionize not only crop selection but also crop creation (Pouvreau et al. 2018). AI accelerates DBTL workflows by predicting gene circuit behaviour, optimizing pathway designs, and improving in-silico simulations before biological implementation. This iterative optimization significantly shortens the time required to engineer crops with traits such as drought tolerance, disease resistance, or enhanced nutritional profiles (Jervis et al. 2019). Recent advances also highlight the use of synthetic biology for engineering plant-associated microbiomes, or synthetic phytobiomass, to improve nutrient acquisition and resilience to stress. AI models are increasingly used to predict microbe–plant interactions, guide the selection of beneficial microbial consortia, and design microbiomes tailored to specific environments or crop genotypes (Compant et al. 2025). Together, these interdisciplinary approaches mark a paradigm shift towards intelligent, predictive, and modular plant engineering frameworks capable of addressing the challenges of 21st-century agriculture.

## Supplementary Information

Below is the link to the electronic supplementary material.

Supplementary file1 (XLSX 30 KB)
